# Sex differences in PD‐L1‐induced analgesia in paclitaxel‐induced peripheral neuropathy mice depend on TRPV1‐based inhibition of CGRP

**DOI:** 10.1111/cns.14829

**Published:** 2024-07-03

**Authors:** Yan Cao, Wenqi Jiang, Fang Yan, Yuyan Pan, Liba Gei, Simin Lu, Xiangnan Chen, Yang Huang, Yan Yan, Yan Feng, Qiang Li, Weian Zeng, Wei Xing, Dongtai Chen

**Affiliations:** ^1^ Department of Anesthesiology, State Key Laboratory of Oncology in South China, Guangdong Provincial Clinical Research Center for Cancer Sun Yat‐Sen University Cancer Center Guangzhou China; ^2^ Department of Anesthesiology Peking University Cancer Hospital (Inner Mongolia Campus)/Affiliated Cancer Hospital of Inner Mongolia Medical University/Inner Mongolia Autonomous Region Cancer Hospital Hohhot China; ^3^ Department of Anesthesiology Guangdong Women and Children Hospital Guangzhou China; ^4^ Department of Anesthesiology Huizhou Municipal Central Hospital Huizhou China

**Keywords:** CGRP, PD‐L1, PIPN, sex differences, TRPV1

## Abstract

**Aims:**

Paclitaxel (PTX) is extensively utilized in the management of diverse solid tumors, frequently resulting in paclitaxel‐induced peripheral neuropathy (PIPN). The present study aimed to investigate sex differences in the behavioral manifestations and underlying pathogenesis of PIPN and search for clinically efficacious interventions.

**Methods:**

Male and female C57BL/6 mice (5–6 weeks and 12 months, weighing 18–30 g) were intraperitoneally (i.p.) administered paclitaxel diluted in saline (NaCl 0.9%) at a dose of 2 mg/kg every other day for a total of 4 injections. Von Frey and hot plate tests were performed before and after administration to confirm the successful establishment of the PIPN model and also to evaluate the pain of PIPN and the analgesic effect of PD‐L1. On day 14 after PTX administration, PD‐L1 protein (10 ng/pc) was injected into the PIPN via the intrathecal (i.t.) route. To knock down TRPV1 in the spinal cord, adeno‐associated virus 9 (AAV9)‐Trpv1‐RNAi (5 μL, 1 × 10^13^ vg/mL) was slowly injected via the i.t. route. Four weeks after AAV9 delivery, the downregulation of TRPV1 expression was verified by immunofluorescence staining and Western blotting. The levels of PD‐L1, TRPV1 and CGRP were measured via Western blotting, RT–PCR, and immunofluorescence staining. The levels of TNF‐α and IL‐1β were measured via RT–PCR.

**Results:**

TRPV1 and CGRP protein and mRNA levels were higher in the spinal cords of control female mice than in those of control male mice. PTX‐induced nociceptive behaviors in female PIPN mice were greater than those in male PIPN mice, as indicated by increased expression of TRPV1 and CGRP. The analgesic effects of PD‐L1 on mechanical hyperalgesia and thermal sensitivity were significantly greater in female mice than in male mice, with calculated relative therapeutic levels increasing by approximately 2.717‐fold and 2.303‐fold, respectively. PD‐L1 and CGRP were partly co‐localized with TRPV1 in the dorsal horn of the mouse spinal cord. The analgesic effect of PD‐L1 in PIPN mice was observed to be mediated through the downregulation of TRPV1 and CGRP expression following AAV9‐mediated spinal cord specific decreased TRPV1 expression.

**Conclusions:**

PTX‐induced nociceptive behaviors and the analgesic effect of PD‐L1 in PIPN mice were sexually dimorphic, highlighting the significance of incorporating sex as a crucial biological factor in forthcoming mechanistic studies of PIPN and providing insights for potential sex‐specific therapeutic approaches.

## Graphical abstract

The sex‐dependent treatment effect of PD‐L1 in PIPN mice was related to the difference in the expression of TRPV1 and CGRP in the spinal cords of PIPN mice of both sexes.

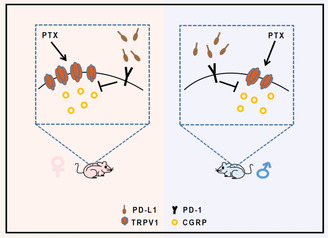

## 1 INTRODUCTION

Paclitaxel (PTX) is widely used to treat various solid tumors, including lung, ovarian, and breast cancers. Although crucial for cancer treatment, PTX use is commonly associated with numerous complications, among which paclitaxel‐induced peripheral neuropathy (PIPN) occurs in 60%–70% of patients undergoing PTX‐based chemotherapy.^1^ The clinical manifestations of PIPN include weakness, numbness, a burning sensation in the limbs, chronic pain and autonomic dysfunction, which seriously affect the quality of life.^2^, ^3^, ^4^ At present, there are no effective clinical drugs available for preventing or treating PIPN, and for patients with neuropathic pain, analgesia is the mainstay and is supplemented with other tranquilizers and nonsteroidal anti‐inflammatory analgesics. Not only are some patients sensitive to drug treatments, but long‐term use of analgesics can also lead to drug resistance and dependence.^5^, ^6^ As research progresses, risk factors for usual chronic pain and acute peripheral neuropathy caused by chemotherapy agents, including age,^7^ sex,^8^ and obesity,^9^ have attracted extensive attention from scholars. Interestingly, a study indicated that postmenopausal patients whose estrogen level decreased are considered to constitute a sub‐population prone to the development of PIPN.^10^ However, less is known about the underlying mechanisms of PIPN and how sex and the aging process are involved.

In recent years, research on sex differences, which are factors that cannot be ignored, in pain has increased dramatically. However, in most of the current research, males are still the most common subjects.^11^ It has been reported that there are significant differences in the incidence rate, clinical manifestations and complications of various diseases between the sexes, and there are even greater differences when stratifying populations based on sex and age. Numerous studies have shown that differences between males and females are more pronounced in pain‐related diseases, such as herpes zoster, headache, backache and multiple chronic pain.^12^, ^13^, ^14^ Moreover, with age‐standardization, headache disorders, oral disorders, and hemoglobinopathies are the greatest causes of morbidity in females.^15^, ^16^, ^17^ In addition to differences in prevalence, pathogenesis and therapeutic efficacy have significant clinical implications. Previous studies have shown that the significant difference in the expression of 44 pain genes is related to sex, and females have approximately twice as many output neurons in the periaqueductal gray‐ rostral ventral medulla (PAG‐RVM) pathway as males.^18^, ^19^ The therapeutic effects of these medications are also sex dependent, and it has been suggested that non‐steroidal anti‐inflammatory drugs are more effective at reducing pain in males than in females, but opioid analgesia such as morphine shows greater efficacy in females.^20^, ^21^ Therefore, sex differences, as a vital factor of PIPN, are the focus of further research. This highlights the necessity of exploring the sex‐dependent and objective pathogenesis of PIPN.

Transient receptor potential vanilloid type 1 (TRPV1), a member of the transient receptor potential (TRP) channel family, is expressed in afferent sensory neurons and is associated with the development of pain.^22^ Excitingly, emerging evidence has shown that spinal administration of a TRPV1 antagonist can prevent and reverse paclitaxel‐induced allodynia. Moreover, inhibition of TRPV1 activity suppressed bone cancer pain through PD‐L1/PD‐1 signaling.^23^, ^24^ Calcitonin gene‐related peptide (CGRP) is a neuropeptide that consists of 37 amino acids generated by selective splicing of the calcitonin gene.^25^ CGRP is widely expressed in the central and peripheral nervous systems, especially in the posterior horn of the spinal cord, which is related to the transmission of pain.^26^, ^27^ Increasing evidence shows that CGRP plays a vital role in the development of peripheral sensitization and associated pain enhancement; most likely, CGRP facilitates the transmission of injury‐related pain. After the injection of capsaicin, TRPV1 activation can induce CGRP release, resulting in the sensitization of primary afferent nociceptors.^28^ However, the exact mechanism by which nociceptive sensitization to PIPN is associated with TRPV1 activation and thus increased CGRP release is unclear, and whether there is a sex difference in PIPN models has not been investigated.

Programmed cell death ligand‐1 (PD‐L1), which is typically synthesized by malignant cells, exerts immunosuppressive effects via interaction with the PD‐1 receptor expressed in T cells.^29^ Previous studies have shown that PD‐L1 is expressed on melanoma and normal nerve cells, including the dorsal root ganglion (DRG), and effectively inhibits acute and chronic pain.^30^ Furthermore, PD‐L1 can also alleviate bone cancer pain and neuropathic pain.^31^, ^32^, ^33^ However, it is not yet known whether PD‐L1 has any analgesic effects in PIPN, and if it does, whether the underlying mechanism is sex‐dependent.

In our study, we established a mouse model of PIPN. Using this model, we confirmed that the PTX‐induced nociceptive behaviors were greater in female mice than in male mice and that these behaviors were related to the differential expression of TRPV1 and CGRP in the spinal cords of both sexes. Additionally, using a TRPV1 knockdown mouse model generated via adeno‐associated virus 9 (AAV9)‐TRPV1‐RNAi virus injection, we investigated the involvement of TRPV1 in PD‐L1‐mediated sex‐specific inhibition of PIPN. This study enhances the understanding of the analgesic effects of PD‐L1 on PIPN and provides insights for potential sex‐specific therapeutic approaches.

## 2 METHODS AND MATERIAL

### 2.1 Animals

Male and female C57BL/6 mice (5–6 weeks and 12 months, weighing 18–30 g) were purchased from the Guangdong Medical Laboratory Animal Centre in Guangzhou, China. All animal experiments were approved by The Sun Yat‐sen University Cancer Centre Animal Care and Use Committee (Sun Yat‐sen University No. L025501202204003). Before performing any experiments, all the mice were quarantined for 7 days. All mice were housed under controlled environmental conditions (temperature: 24 ± 1°C, humidity: 50%–60%) and had a 12‐h light/dark cycle with water and food available ad libitum.

### 2.2 Treatment of animals

To induce PIPN pain, male and female C57BL/6 mice were i.p. administered PTX diluted in saline (NaCl 0.9%) at a dose of 2 mg/kg every other day for a total of 4 injections (days 0, 2, 4, and 6).^34^, ^35^, ^36^ The control group was i.p. administered the same volume of physiological saline. Von Frey and hot plate tests were performed on days 1, 3, 5, 7, 14 and 21 after injection to confirm the successful establishment of the PIPN models.

For i.t. injection of the PD‐L1 protein (diluted in saline, 10 ng or sterile water) on day 14 after PTX injection, spinal cord puncture between the L5 and L6 levels was performed with a Hamilton syringe equipped with a 30‐gauge needle to deliver reagents.^37^ The accuracy of the i.t. injection was confirmed by observing the tail flick response in the mice.

### 2.3 Intrathecal injection of AAV virus

To functionally determine the role of TRPV1 in the sex‐dependent analgesic effect of PD‐L1 in PIPN, we used RNA interference driven by AAV9‐Trpv1‐RNAi to downregulate TRPV1 levels in the spinal cord. To knock down TRPV1, we selected the siRNA sequences GGTACTGTACTTCAGCCATCG and GCAACTGTGAGGGC GTCAAGC to construct AAV viral vectors (Gene, China). After the mice were anesthetized with pentobarbital, a 30‐gauge needle connected to a Hamilton syringe was introduced into the L5‐ and L6‐level space. Sudden tail movement indicated successful delivery of the catheter. Subsequently, the virus solution (5 μL, 1 × 10^13^ vg/mL) was injected slowly, and the animals were restricted from movement for a minimum of 1 min following injection to prevent drug efflux. Four weeks after AAV9 delivery, the downregulation of TRPV1 expression was verified by immunofluorescence staining and Western blotting.

### 2.4 Measurement of mechanical hyperalgesia using the Von‐Frey test

Prior to commencing the formal experiment, we acclimated individual mice under glass on an elevated wire mesh grid for 1 h per day for a total of 3 days to facilitate adaptation to the experimental environment. Subsequently, we employed a series of Von Frey hairs with logarithmically increasing stiffness (ranging from 0.04 to 2.0 g) to evaluate the paw withdrawal threshold. A positive response was defined as either hind paw licking or rapid withdrawal. Once the back paw has shown a positive response in at least 3 out of 5 measurements at 5‐min intervals for both the left and right hind paws, the fiber value in grams is considered PWT.^24^, ^38^

### 2.5 Measurement of thermal sensitivity using hot plate tests

Thermal sensitivity (paw withdrawal latency) was determined using a hot plate set (ZH‐6C, ANHUI Zenna Biologenic Apprax FACILITIES, China) at 55°C 2 h after the von Frey test. Before the formal experiments, mice were subjected to an adaptive test for 3 days, as previously described.^39^ Paw licking, paw contraction, scratching or four‐foot hopping were considered positive responses. The endpoint was this reaction time, and an increase in hot plate delay was used to determine the analgesic activity. In order to prevent tissue injury, mice were promptly removed from the hot plate if they did not respond within 30 s. The thermal sensitivity test was conducted three times at 5‐min intervals, and the average value was calculated.

### 2.6 Western blotting

After deep anesthesia, L4‐L5 spinal dorsal horn tissue segments were collected from PIPN mice and control mice and subsequently frozen at −80°C until required for further use. The proteins were lysed with RIPA buffer, and the concentrations of the lysates were measured via a BCA protein assay as described previously.^37^ Equal amounts of proteins were separated via 10% or 12.5% SDS–PAGE and transferred to polyvinylidene fluoride (PVDF) membranes. The membranes were blocked with Tris‐buffered saline containing Tween‐20 (TBST) and containing 5% nonfat dry milk (FUJIFILM Wako, Japan) or BSA (Epizyme Biotech, China) for 1 h at room temperature and then incubated with primary antibodies against GAPDH (1:10,000), PD‐L1 (1:500‐1:1000), TRPV1 (1:1000), and CGRP (1:500‐1:1000) overnight at 4°C. On the second day, the membranes were washed and incubated with species‐matched secondary antibodies at room temperature for 1 h. The protein bands were visualized using Millipore's enhanced chemiluminescence reagent. All Western blot experiments were conducted in triplicate, and the integrated optical density of the bands was quantified using a Bio‐Rad image analysis system (Bio‐Rad Laboratories, USA) and ImageJ software.

### 2.7 Quantitative RT–PCR

The L4–L5 segments of spinal dorsal horn tissue were collected, and RNA was extracted using a Tissue RNA Purification Kit Plus (ES Science, China) according to the manufacturer's instructions. cDNA synthesis was performed using an EZBioscience Color Reverse Transcription Kit (Roseville, USA). RT–PCR was performed with SYBR Green qPCR Super Mix (EZBioscience, Roseville, USA) on a CFX96 Touch Real‐Time PCR System (Bio‐Rad, USA), and the results were calculated by the comparative threshold cycle (Ct) method. Assays were conducted in technical replicates, and the results were normalized to mouse GAPDH as a reference standard.

### 2.8 Immunofluorescence staining

The mice were anesthetized via i.p. injection of sodium pentobarbital (100 mg/kg) and subsequently perfused through the heart with PBS followed by 4% cold paraformaldehyde. The segments were subsequently immersed in a 30% sucrose solution at a temperature of 4°C until they sunk to the bottom of the container following fixation. The samples were sectioned into 25 μm thick sections on a cryostat and fixed on gelatin‐coated glass slides as described previously.^40^ The sections were blocked with Immunol Staining Blocking Buffer (Beyotime, China) at room temperature for 1 h and subsequently incubated overnight with rabbit anti‐PD‐L1 (1:100–1:250) and mouse anti‐TRPV1 (1:100–1:200) primary antibodies mixed with anti‐NeuN (1:200) at 4°C. After washing, the sections were incubated with FITC‐Alexa Fluor 488‐ and Cy3‐conjugated secondary antibodies (1:500–1:1000) at room temperature for 1 h. Sections were viewed using a confocal laser scanning microscope (LSM980, Zeiss, Germany). The data were analyzed with ImageJ software.

### 2.9 Hematoxylin–eosin staining

The mice were euthanized at a designated time. The hearts, livers, spleens, lungs and kidneys were collected, fixed with para‐formaldehyde, and then stained with hematoxylin and eosin (H&E).

Antibodies and oligos used for Western blot, immunofluorescence, RT–PCR, and AAV–RNAi assays were showed in Table 1.

**TABLE 1:** Antibodies and oligoes used for Western blot, IF, RT‐PCR, and AAV‐RNAi assays in this study.

Reagent or Resource	Source	Identifier
Antibodies/Protein		
Recombinant Mouse PD‐L1 protein	Abcam	Cat# ab130039
Rabbit monoclonal anti‐PD‐L1	Abcam	Cat# ab213480
Mouse monoclonal anti‐TRPV1	Abcam	Cat# ab203103
Rabbit monoclonal anti‐TRPV1	Abcam	Cat# ab305299
Rabbit monoclonal anti‐CGRP	Cell Signaling Technology	Cat# ab14959
Mouse monoclonal anti‐GADPH	GeneTex	Cat# GT293
Donkey anti‐mouse IgG Cy3	Jackson ImmunoResearch	Code:715–165‐151
Goat anti‐Rabbit IgG‐488	ThermoFisher	Cat# A‐11008
Goat anti‐mouse IgG‐488	ThermoFisher	Cat# A‐11001
Oligonucleotides		
Mouse PD‐L1‐F		TGCGGACTACAAGCGAATCACG
Mouse PD‐L1‐R		CTCAGCTTCTGGATAACCCTCG
Mouse TRPV1‐F		CATCTTCACCACGGCTGCTTAC
Mouse TRPV1‐R		CAGACAGGATCTCTCCAGTGAC
Mouse CALCA‐F		GCACTGGTGCAGGACTATATGC
Mouse CALCA‐R		CTCAGATTCCCACACCGCTTAG
Mouse TNF‐a‐F		GGTGCCTATGTCTCAGCCTCTT
Mouse TNF‐a‐R		GCCATAGAACTGATGAGAGGGAG
Mouse IL‐1b‐F		TGGACCTTCCAGGATGAGGACA
Mouse IL‐1b‐R		GTTCATCTCGGAGCCTGTAGTG
Mouse GADPH‐F		CATCACTGCCACCCAGAAGACTG
Mouse GADPH‐R		ATGCCAGTGAGCTTCCCGTTCAG
siRNA Target Seq		
TRPV1‐siRNA 2		GCCTGAAGCAGTTTGTCAATG
TRPV1‐siRNA 3		GGTACTGTACTTCAGCCATCG
TRPV1‐siRNA 4		GCAACTGTGAGGGCGTCAAGC

Synthetic oligo information					
NO.	5′	STEM	Loop	STEM	3′
Trpv1‐RNAi(2–1)‐a	ACCGG	GCCTGAAGCAGTTTGTCAATG	TTCAAGAGA	CATTGACAAACTGCTTCAGGC	TTTTT
Trpv1‐RNAi(2–1)‐b	TCTAAAAAA	GCCTGAAGCAGTTTGTCAATG	TCTCTTGAA	CATTGACAAACTGCTTCAGGC	C
Trpv1‐RNAi(3–1)‐a	ACCGG	GGTACTGTACTTCAGCCATCG	TTCAAGAGA	CGATGGCTGAAGTACAGTACC	TTTTT
Trpv1‐RNAi(3–1)‐b	TCTAAAAAA	GGTACTGTACTTCAGCCATCG	TCTCTTGAA	CGATGGCTGAAGTACAGTACC	C
Trpv1‐RNAi(4–1)‐a	ACCGG	GCAACTGTGAGGGCGTCAAGC	TTCAAGAGA	GCTTGACGCCCTCACAGTTGC	TTTTT
Trpv1‐RNAi(4–1)‐b	TCTAAAAAA	GCAACTGTGAGGGCGTCAAGC	TCTCTTGAA	GCTTGACGCCCTCACAGTTGC	C

### 2.10 Statistical analysis

For all experiments, the number of biological replicates (*n*), measure of central tendency (e.g. average), error bars, and statistical analysis has been explained in the Figure legends. For each experiment where statistics were computed, we used at least *n* = 3 or more biological replicates. All statistically significant comparisons are detailed in the Figures and corresponding legends. Data are presented as mean ± SEMs after testing for normality by Shapiro–Wilk test using GraphPad Prism software (version 9, GraphPad Software, San Diego, CA, USA). Unpaired two‐tailed Student *t* test is used to compare the means of 2 groups. To compare multiple groups, comparisons of the means are performed using one‐way or two‐way ANOVA followed by the Bonferroni post hoc correction to determine statistically significant differences. A *p* value of <0.05 was considered to indicate statistical significance.

## 3 RESULTS

### 3.1 Greater PTX‐induced nociceptive behavior was observed in female mice than in male mice

**FIGURE 1:**
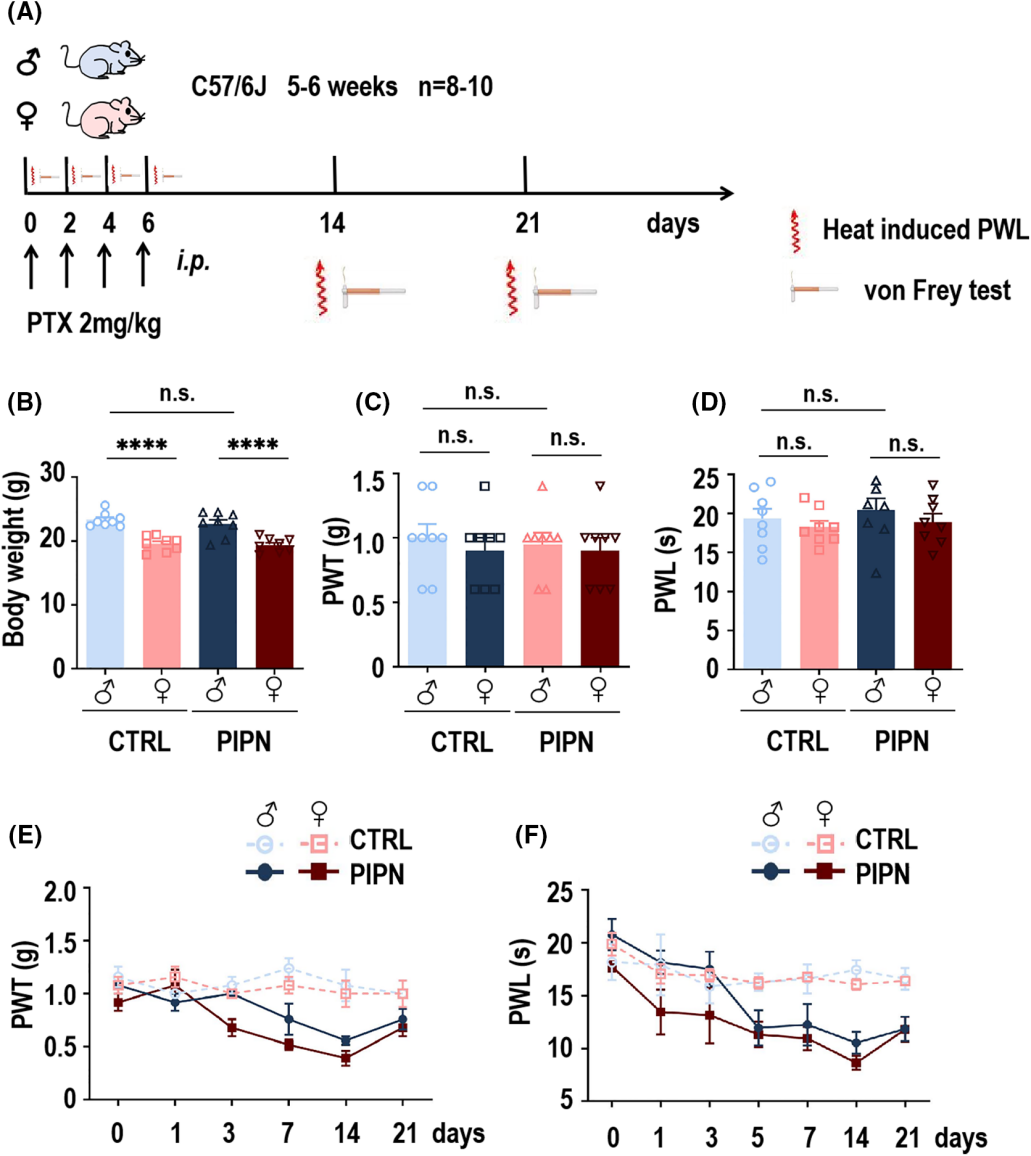
Paclitaxel (PTX) induced sex differences in pain‐related behaviors in mice. (A) Schematic diagram of the process of behavioral testing in the PTX‐induced paclitaxel‐induced peripheral neuropathy (PIPN) model. Both sexes of mice (C57/6 J, 5–6 weeks, *n* = 8–10 per group) were conditioned with injections of PTX (2 mg/kg, i.p., 4 times) or the saline agent. Pain‐like behaviors were tested after each treatment and after 14 and 21 days for each group. (B) Body weights of both the control and PIPN mice of both sexes before PTX injection. The results are represented as Mean ± SEM. ****p < 0.0001, n.s., not statistically significant difference (One‐way ANOVA). (C, D) Mechanical allodynia (C) and thermal hyperalgesia (D) for both control and PIPN mice of both sexes before PTX treatment. The results are represented as Mean ± SEM. n.s., not statistically significant difference (One‐way ANOVA). (E, F) PTX (2 mg/kg, four times, i.p.) induced sexually dysmorphic significant mechanical allodynia (E) and thermal hyperalgesia (F) using two‐way ANOVA analysis in the hind paw after 14 days.

To investigate whether the effects of PTX differed based on the sex of the mice, we first established a PIPN mouse model via four i.p. injections of 2 mg/kg PTX once a day. Behavioral tests of mechanical hyperalgesia and thermal sensitivity were performed on days 1, 3, 5, 7, 14 and 21 after injection (schematic diagram shown in Figure 1A). The body weights of 5‐6‐week‐old male and female C57/6J mice used for PIPN model establishment significantly differed; however, there was no statistical difference between the weights of control and PIPN mice within the male or female groups (Figure 1B). Prior to PTX injection, there was no statistically significant difference in mechanical hyperalgesia and thermal sensitivity in control and PIPN group mice of both sexes (*n* = 8–10, 5–6 weeks; Figure 1C,D). The paw withdrawal threshold (PWT) (Figure 1E) and paw withdrawal latency (PWL) (Figure 1F) decreased more in female mice than male mice and both significantly differed from those observed in the control group on days 7 and 14. Moreover, PWT and PWL recovered gradually after 21 days.

**FIGURE 2:**
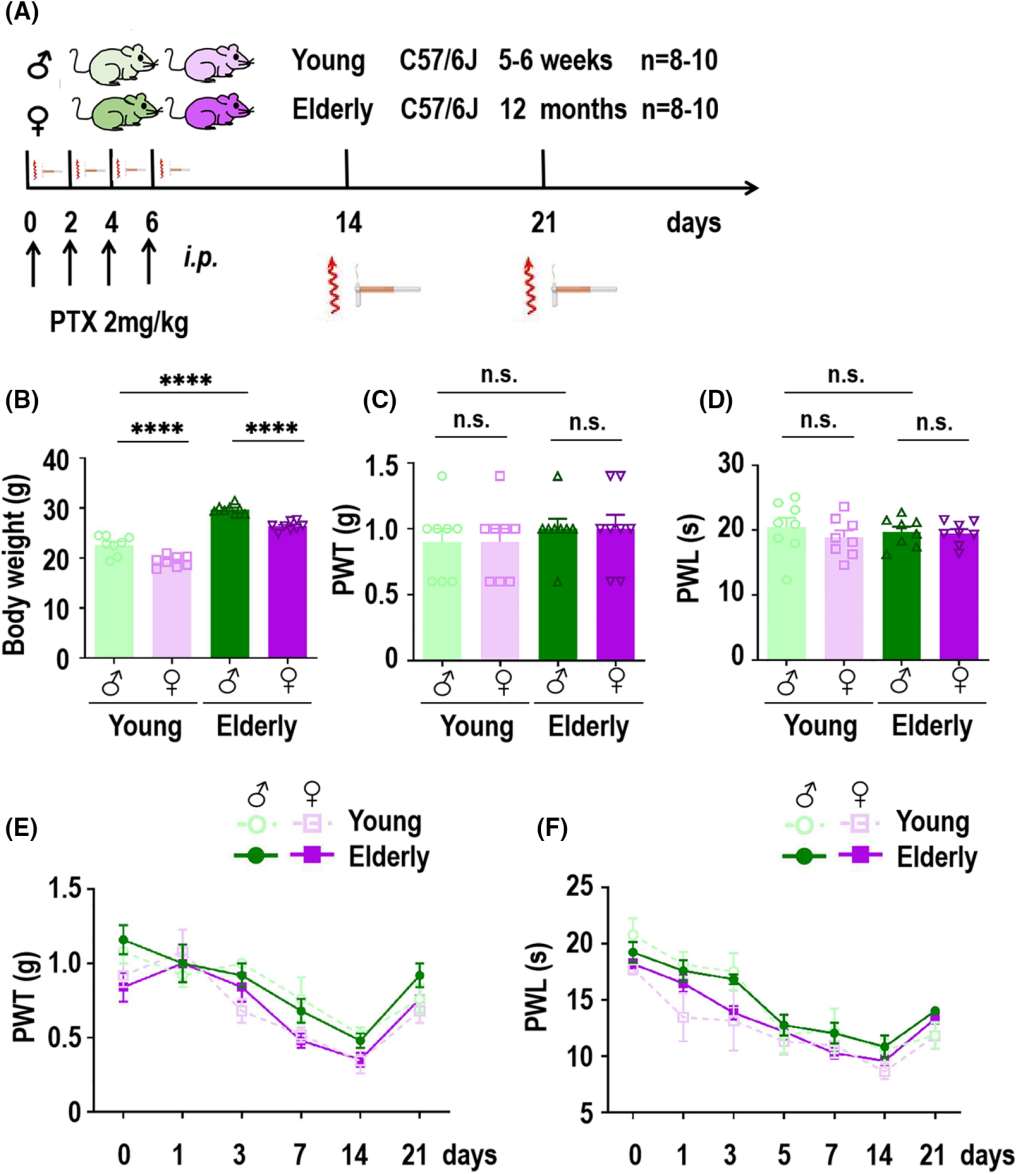
Paclitaxel (PTX) induced sex differences in pain‐related behaviors in elderly mice. (A) Schematic diagram of the behavioral test in the PIPN model. Both the young and elderly mice (C57/6J, 5–6 or 12 months; *n* = 8–10 per group) were conditioned with injections of PTX (2 mg/kg, i.p., 4 times). Pain‐like behaviors were tested after each treatment and after 14 and 21 days for each group. (B) Body weight of young and elderly PIPN mice before PTX treatment. The results are represented as Mean ± SEM. ****p < 0.0001, n.s., not statistically significant difference (One‐way ANOVA). (C, D) Mechanical allodynia (C) and thermal hyperalgesia (D) for both sexes of young and elderly PIPN mice before PTX treatment. The results are represented as Mean ± SEM. n.s., not statistically significant difference (One‐way ANOVA). (E, F) PTX (2 mg/kg, 4 times, i.p.) induced no changes in significant mechanical allodynia (E) or thermal hyperalgesia (F) using two‐way ANOVA analysis in the hind paw after 14 days.

To further explore whether sex differences in PTX‐induced pain‐related behaviors were age‐related, we next established young and elderly PIPN mouse models via the same protocol as above (schematic diagram shown in Figure 2A). The body weights of male and female C57/6J mice aged 5–6 weeks used to establish the young PIPN mouse mode significantly different, and statistical differences in the weights of young and elderly mice was observed in both the male and female groups were observed (Figure 2B). Before modeling, there was no statistically significant difference in the PWT or PWL between the young and elderly groups of either sex (*n* = 8–10, 5–6 weeks vs. 12 months; Figure 2C,D). The behavioral experiment result revealed that the PWT (Figure 2E) and PWL (Figure 2F) also decreased more in young or elderly female mice than in the corresponding male mice on days 7 and 14. However, there was no difference between young and elderly female or male mice. Consistent with previous results, all four groups recovered gradually after 21 days. Taken together, these findings provide strong evidence that PTX induced sex‐difference pain‐related behaviors in mice and we explored the underlying mechanism further.

### 3.2 The analgesic effect of PD‐L1 in PIPN mice was sexually dimorphic

**FIGURE 3:**
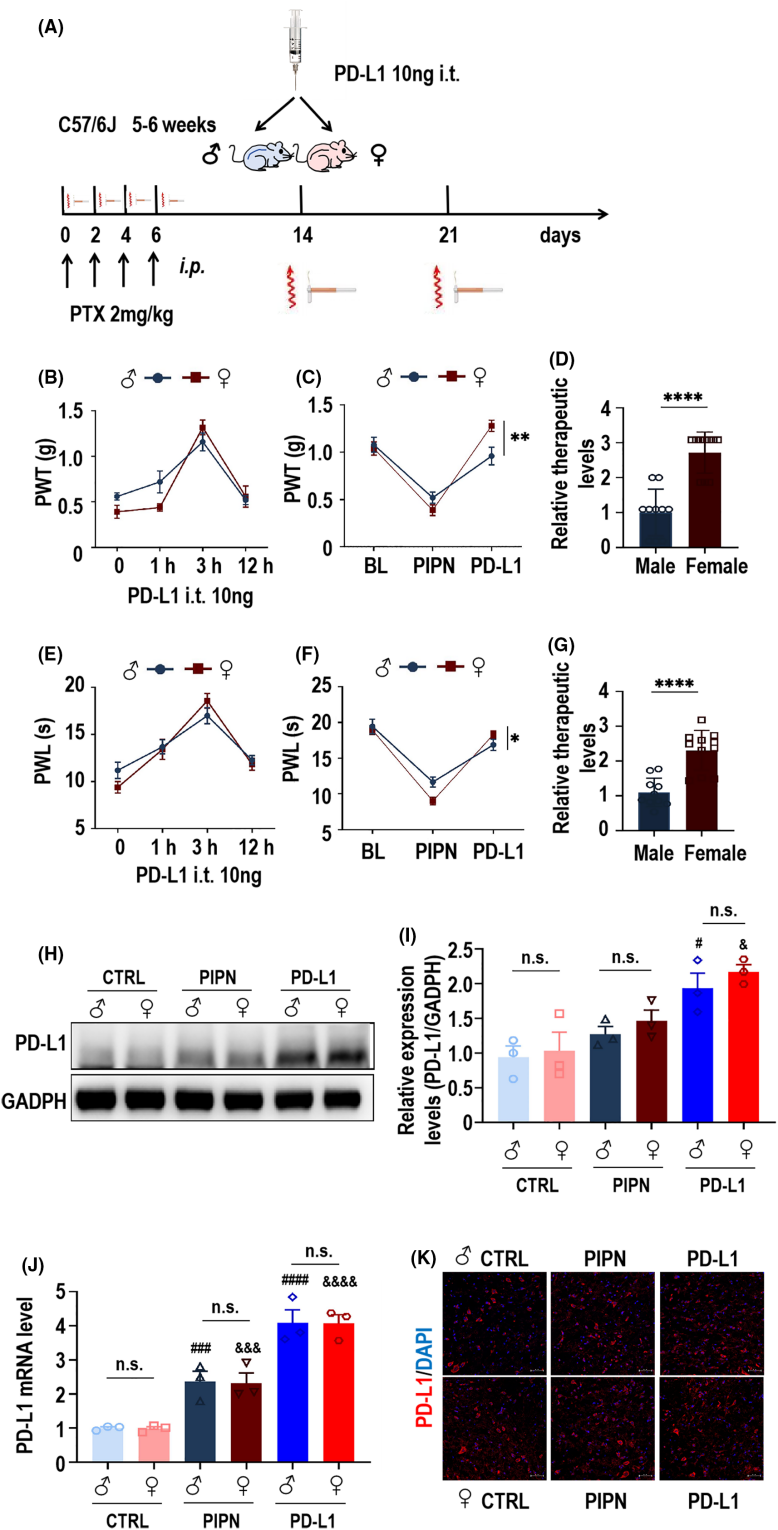
Involvement of PD‐L1 in sex differences in the PIPN mice. (A) Schematic diagram of the process of behavioral testing in the PTX‐induced PIPN model. Both sexes of mice (C57/6J, 5–6; *n* = 8–10 per group) were induced by PTX (2 mg/kg, i.p., 4 times). PD‐L1 (10 ng) was injected into both sexes of PIPN mice on day 14 by i.t. Pain‐like behaviors were tested on days 1, 3, 5, 7, 14 and 21 for each group. (B) Mechanical allodynia was measured at 1, 3 and 12 h after PD‐L1 treatment. (C) PD‐L1‐induced significant mechanical allodynia was alleviated at 3 h after treatment in a sex‐dependent manner using two‐way ANOVA analysis. **p < 0.01. (D) Calculated relative therapeutic mechanical allodynia levels in both sexes of PIPN mice. The data are presented as the means ± SEMs. ****p < 0.0001 (two‐tailed Student's *t‐*test). (E) Thermal hyperalgesia was measured at 1, 3 and 12 h after PD‐L1 treatment. (F) PD‐L1 alleviated PTX‐induced significant thermal hyperalgesia at 3 h after treatment in a sex‐dependent manner using two‐way ANOVA analysis. *p < 0.05. (G) Calculated relative therapeutic thermal hyperalgesia levels resulting from PD‐L1 treatment in both sexes of PIPN mice. The data are presented as the means ± SEMs. ****p < 0.0001 (two‐tailed Student's *t‐*test). (H–J) WB results (H, I) or qPCR results (J) for detecting PD‐L1 expression in the spinal cords of both sexes control, PIPN and PIPN groups treated with PD‐L1 for 3 h using two‐way ANOVA analysis. ^#^ *p* < 0.05, ^###^ *p* < 0.001 and ^####^ *p* < 0.0001 compared with the male control group. ^&^ *p* < 0.05, ^&&&^ *p* < 0.001 and ^&&&&^ *p* < 0.0001 compared with the female control group. n.s., not statistically significant difference. (K) Immunofluorescence images showing PD‐L1 expression (red) in the spinal cords of both sexes control, PIPN and PIPN groups treated with PD‐L1. Nuclei were labeled with DAPI (blue). Scale bar: 50 μm.

To assess the potential analgesic effect of PD‐L1 in male and female PIPN mice, we injected 10 ng of PD‐L1 into the spinal cord of PIPN mice on day 14. The details are shown in Figure 3A. To further evaluate the optimal time of assessing the PD‐L1 effect, we measured the PWT (Figure 3B) and PWL (Figure 3E) in mice of both sexes before injection and at 1, 3, and 12 h after PD‐L1 injection. As expected, we found that the analgesic effect of PD‐L1 was optimal 3 h after injection. Next, we focused on calculating mechanical hyperalgesia and thermal sensitivity at 3 h after injection. The rescue effect of PD‐L1 on PWT was significantly greater in female mice than in male mice (Figure 3C), and the calculated relative therapeutic effect was almost 2.717‐fold higher in the female PIPN group than in the male PIPN group (Figure 3D). Simultaneously, PD‐L1 had the same therapeutic effect on PWL in mice, and the effect was also significantly greater in female mice than in male mice (Figure 3F). The relative therapeutic effect on the PWL was approximately 2.303‐fold higher in the female PIPN group than in the male PIPN group (Figure 3G). Notably, we observed a robust increase in the protein (Figure 3H,I) and mRNA (Figure 3J) levels of PD‐L1 after injection into the spinal cords of both sexes. Consistently, immunofluorescence staining also showed that increased expression of PD‐L1 in the spinal cords of PIPN mice of both sexes (Figure 3K). These results demonstrated that the involvement of PD‐L1 in PIPN mice was sexually dimorphic. In order to assess the potential toxic effects on mice in each experimental group, we conducted continuous measurements of body weight from pre‐experiment baseline through 14 days after PTX injection and PD‐L1‐treatment. Our findings indicated that there were no significant differences in body weight among the PIPN and PD‐L1 groups compared to the control group within their respective genders (Figure S1A). The histological analysis of the heart, liver, spleen, lung and kidney revealed no apparent pathological changes following treatment compared to both sexes control groups respectively (Figure S1B). This further indicated that the experimental procedure did not induce acute toxicity or tissue damage and there was no discernible difference between males and females.

### 3.3 Role of TRPV1 and CGRP in the analgesic effect of PD‐L1 in both sexes of PIPN mice

**FIGURE 4:**
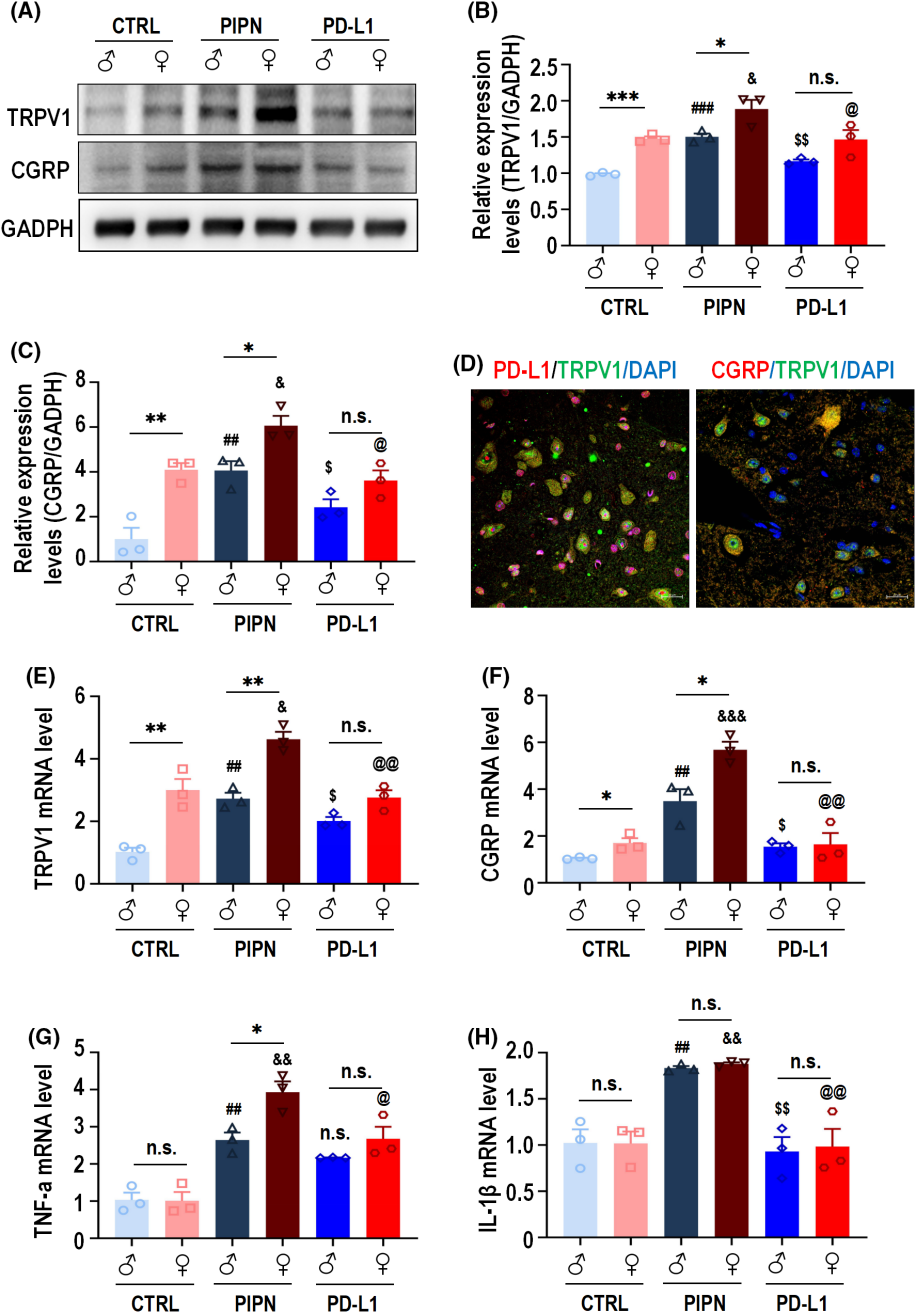
Dynamic changes in TRPV1 and CGRP expression in the spinal cord after PTX injection and the effects of PD‐L1 on TRPV1 function. (A–C) WB analysis of TRPV1 (A, B) and CGRP (A, C) expression in the spinal cords of both sexes control, PIPN and PIPN groups with 3h‐PD‐L1‐treatment using two‐way ANOVA analysis. *p < 0.05, **p < 0.01, ***p < 0.001 compared with the male control or PIPN group. ^##^ *p* < 0.01, and ^###^ *p* < 0.001 compared with the male control group. ^&^ *p* < 0.05 compared with the female control group. ^$^ *p* < 0.05, and ^$$^ *p* < 0.01 compared with the male PIPN group. ^@^ *p* < 0.05 compared with the female PIPN group. n.s., not statistically significant difference. (D) Representative images of double‐staining for PD‐L1 (red) and TRPV1 (green) in the spinal cords of PIPN mice on day 14. Representative images of double‐staining for CGRP (red) and TRPV1 (green) in the spinal cords of PIPN mice on day 14. Nuclei were labeled with DAPI (blue). Scale bar: 20 μm. (E–H) qPCR results for detecting TRPV1 (E), CGRP (F), TNF‐a (G) and IL‐1β (H) expression in the spinal cords of both sexes control, PIPN and PIPN mice treated with PD‐L1 for 3 h using two‐way ANOVA analysis. *p < 0.05 and **p < 0.01 versus the male control or PIPN group. ^##^ *p* < 0.01 versus the male control group. ^&^ *p* < 0.05, ^&&^ *p* < 0.01 and ^&&&^ *p* < 0.0001 versus the female control group. ^$^ *p* < 0.05, and ^$$^ *p* < 0.01 versus the male PIPN group. ^@^ *p* < 0.05 and ^@@^ *p* < 0.01 versus the female PIPN group. n.s., not statistically significant difference.

To understand how PD‐L1 plays an analgesic role, we investigated whether certain proteins related to pain are affected by PD‐L1 upregulation in PIPN mice. To this end, we examined the expression of TRPV1 (which plays an important role in the PIPN mouse DRG^41^) in the spinal cords of the mice in each group by Western blot analysis. Treatment with PTX increased the levels of TRPV1 and CGRP in the spinal cords of mice of both sexes (Figure 4A, lane 3 vs. 1, lane 4 vs. 2; Figure 4B). Importantly, we found that the basal expression levels of TRPV1 and CGRP were significantly greater in the female spinal cord than in the male spinal cord (Figure 4A, lane 2 vs. 1; Figure 4B). The addition of PD‐L1 for 3 h partly blocked the PTX‐induced upregulation of TRPV1 and CGRP at the protein level in the spinal cords of mice of both sexes (Figure 4A, lane 5 vs. 3; lane 6 vs. 4) as well as at the mRNA levels (TRPV1 [Figure 4E] and CGRP [Figure 4F]). Meanwhile, immunofluorescence staining showed that PD‐L1, CGRP and TRPV1 were partially co‐localized in the dorsal horn of PIPN mice spinal cord on day 14 (Figure 4C). We also detected the mRNA levels of the inflammatory cytokines TNF‐α (Figure 4G) and IL‐1β (Figure 4H), the results of which were consistent with those described above. Collectively, these results indicate that, in PIPN mice, the analgesic effect of PD‐L1 is sexually dimorphic and might occur through TRPV1 and CGRP.

### 3.4 TRPV1 is involved in PD‐L1‐induced sex differences in PIPN inhibition

**FIGURE 5:**
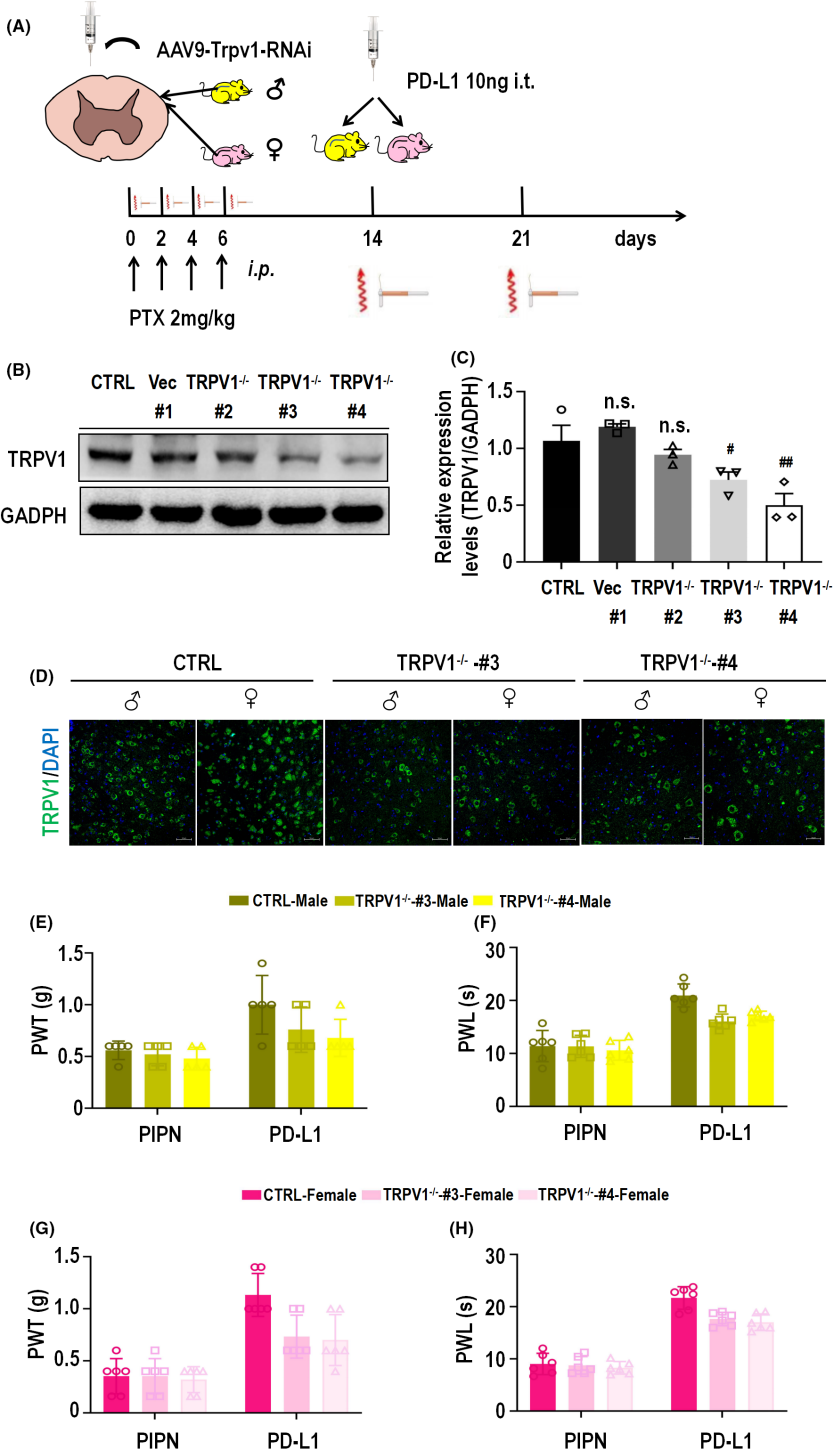
Knockdown of TRPV1 expression in the spinal cord blocks the PD‐L1‐induced analgesic effect in both sexes of PIPN mice. (A) A schematic diagram of the behavioral test of TRPV1^−/−^mice infected with AAV9‐TRPV1‐RNAi virus (sequences are plotted in Table 1). Mice of both sexes were conditioned with injections of PTX (2 mg/kg, i.p., 4 times) or PD‐L1 (10 ng, i.t.) on day 14. (B, C) WB results for detecting TRPV1 expression in the spinal cord of male control, PIPN and PIPN with PD‐L1 treatment for 3 h groups using two‐way ANOVA analysis are shown. ^#^ *p* < 0.05 and ^##^ *p* < 0.01 versus the male control group. (D) Immunofluorescence staining images showing TRPV1 expression (green) in the spinal cords of both sexes control, PIPN and PIPN‐treated PD‐L1 group mice. Nuclei were labeled with DAPI (blue). Scale bar: 50 μm. (E) Knocking down TRPV1 expression in the spinal cord partly blocked PD‐L1‐induced significant mechanical allodynia in male PIPN mice. (F) Knocking down TRPV1 expression in the spinal cord partly blocked PD‐L1‐induced PTX‐induced significant thermal hyperalgesia in male PIPN mice. (G) Knocking down TRPV1 expression in the spinal cord blocked PD‐L1‐induced significant mechanical allodynia in female PIPN mice. (H) Knocking down TRPV1 expression in the spinal cord blocked PD‐L1‐induced significant thermal hyperalgesia in female PIPN mice.

**FIGURE 6:**
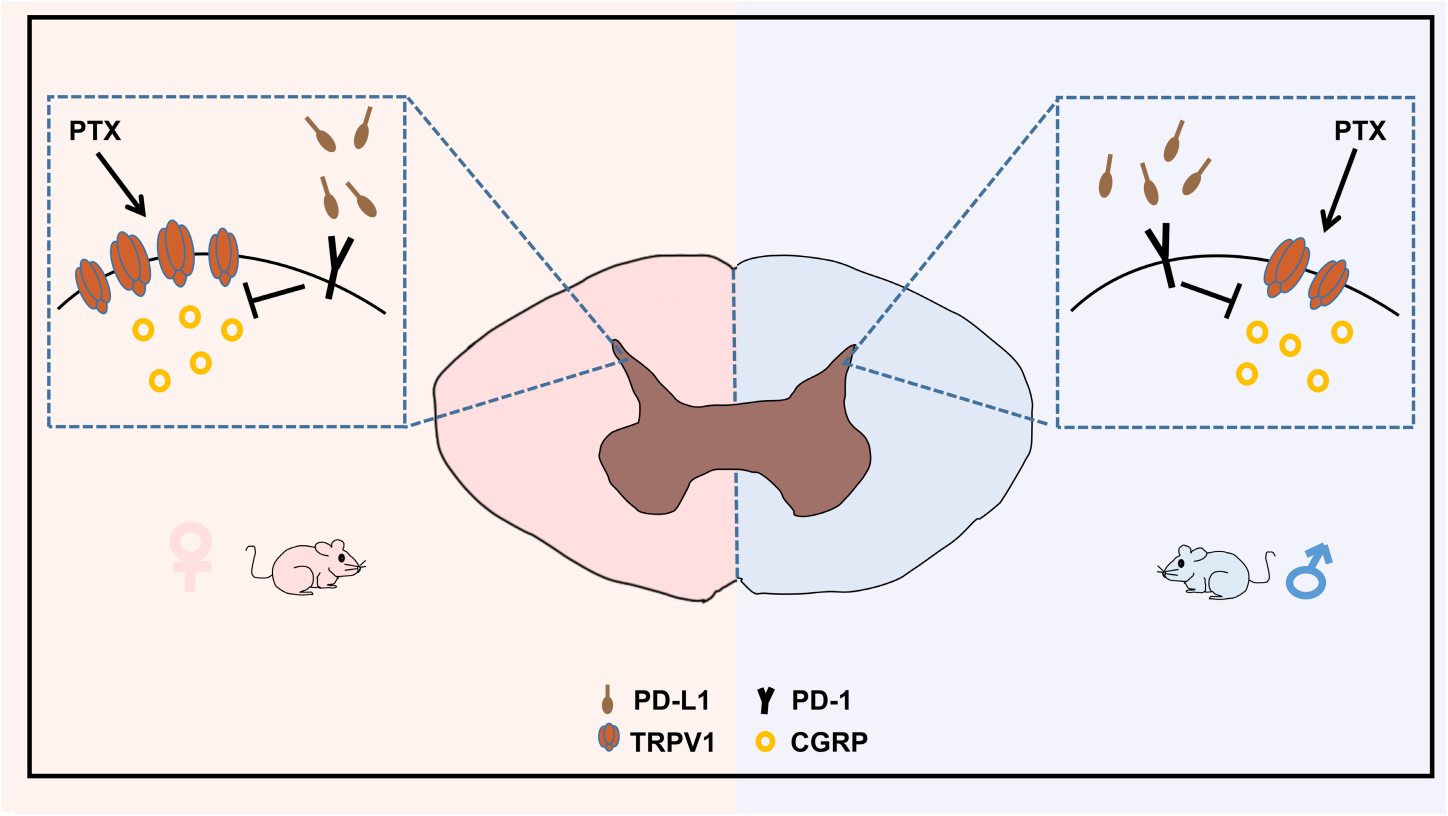
A schematic diagram of the sex‐dependent PD‐L1‐induced analgesic effect on TRPV1. PTX‐induced nociceptive behaviors and PD‐L1‐induced analgesic effects were greater in female mice than in male mice. PD‐L1 suppressed TRPV1 and CGRP expression in the spinal cords of PIPN mice.

The above experimental results showed that the involvement of PD‐L1 in PIPN mice was sexually dimorphic and that injection of PD‐L1 could partially block the PTX‐induced upregulation of TRPV1 and CGRP at the protein and mRNA levels in the spinal cords of both male and female mice. However, the necessity of TRPV1 for the analgesic effect of PD‐L1 on PIPN has not been determined. We generated a TRPV1 knockdown mouse model by i.t. injection of vector #1 or AAV9‐TRPV1‐RNAi viruses #2, #3 and #4. Next, PTX and PD‐L1 were administered to mouse models as described above (Figure 5A). Western blot analysis (Figure 5B,C) revealed that TRPV1 knockdown by AAV9‐TRPV1‐RNAi virus #3 and #4 was significant (significant difference) after 4 weeks of injection. These constructs were used for subsequent experiments. Consistent with the Western blot results, compared with those of the control group, immunofluorescence staining of spinal cord sections from TRPV1^−/−^‐#3 and #4 mice of both revealed a significant decrease in TRPV1 expression. Notably, TRPV1 expression in the spinal cord was markedly greater in the control female group than in the corresponding male group. Moreover, there was no significant difference in the expression of TRPV1 between female and male TRPV1^−/−^#3 or TRPV1^−/−^#4 mice (Figure 5D). After PD‐L1 injection (10 ng) for 3 h on day 14, the analgesic effect of PD‐L1 on the male TRPV1^−/−^‐#3 and ‐#4 mouse models, as measured by the PWT and PWL, was partly blocked compared with that on the control group (Figure 5E,F). Similar results were obtained for the female TRPV1^−/−^ model (Figure 5G,H). Moreover, there was no significant difference in the analgesic effect of PD‐L1 between female and male TRPV1^−/−^ mice. Collectively, these findings provide strong evidence that TRPV1 is involved in the PD‐L1‐induced sex difference inhibition of PIPN, which is shown in the schematic diagram in Figure 6.

## 4 DISCUSSION

As a coinhibitory checkpoint molecule, PD‐L1 has been shown to exert suppressive effects on T‐cell responses within the tumor microenvironment. The primary clinical application of PD‐L1 is immunotherapy in cancer patients.^42^ However, emerging research has elucidated the specific mechanisms by which PD‐L1 is involved in pain development and its analgesic effects. It has been suggested that the PD‐L1/PD‐1 signaling pathway can exert analgesic effects on bone cancer pain in mice by inhibiting TRPV1 activity in primary sensory neurons via SHP‐1 to delay pain or by suppressing PD‐L1/CCl2‐mediated osteoclastogenesis and protecting against bone destruction.^24^, ^31^ However, it remains unclear whether PD‐L1 regulating TRPV1 expression plays an analgesic role in PIPN, and if so, the underlying mechanism and potential sex‐dependent effects have yet to be elucidated. In our study, we successfully established both male and female PIPN models and observed that female mice exhibited more pronounced mechanical allodynia and thermal hyperalgesia than did male mice. Excitingly, the female mice showed better analgesic effect by PD‐L1 via inhibiting TRPV1 and CGRP expressions.

Furthermore, we obtained mouse spinal cords to conduct a more comprehensive investigation of PD‐L1. Some studies have postulated that PD‐L1 can serve as an endogenous pain inhibitor and that PD‐L1 is widely expressed in neurons.^43^, ^44^ We subsequently validated these observations by double immunofluorescence staining. We also observed the widespread distribution of PD‐L1 in the dorsal horn of the mouse spinal cord. Our IF results revealed that PD‐L1 was expressed in the cell membrane, cytoplasm, and nucleus, consistent with previous studies.^45^ Researchers propsed the term “spatial heterogeneous expression” to describe the varying expression of PD‐L1 in different subtypes of cells, including cytoplasm, nucleus, and even exosomes. This finding also distributed to elucidate the limitations of current approaches to PD‐L1 targeted therapies. Furthermore, several scholars had demonstrated that the nuclear translocation of PD‐L1 could enhance the angiogenesis of malignant tumors.^46^ Similarly, it is noteworthy that in our PIPN model, there was a significant increase in the expression of PD‐L1 in the nucleus. Therefore, we hypothesized that nuclear translocation might also occur in PD‐L1 during the development of PIPN, which were needed to verify through rigorous experimental design in the future. This also provided new insights for further studying the heterogeneity of PD‐L1 expression and the mechanism of nuclear translocation of PD‐L1 in the analgesia effect of PIPN.

TRPV1 expression is widely recognized to increase following painful and high‐temperature stimuli.^47^ TRPV1 is expressed in afferent sensory neurons, whose bodies transmit information about environmental stimuli to the central nervous system through the dorsal horn of the spinal cord.^48^, ^49^ Growing evidence suggests that CGRP, which is widely expressed in the trigeminal nucleus caudalis and spinal cord dorsal horn, plays an important role in the development of peripheral sensitization and associated pain enhancement. CGRP can promote the development of neurogenic inflammation, and CGRP expression is upregulated in the presence of neuropathic pain and inflammation.^50^, ^51^, ^52^ The protein and mRNA levels of CGPR in the spinal dorsal horn and dorsal root ganglia are upregulated in chronic and osteoarthritis pain models.^53^, ^54^, ^55^ Moreover, some studies have clearly concluded that when TRPV1 C‐fibers in the pancreas are stimulated, CGRP is released, resulting in neurogenic inflammation. TRPV1‐mediated CGRP release may also be associated with migraine headaches.^56^, ^57^ In other studies, in which hyperalgesia and allodynia models were constructed, the release of CGRP in dorsal spinal cord sections increased in response to capsaicin treatment.^58^, ^59^ In summary, the expression of TRPV1 and CGRP and the regulatory relationship between these molecules play important roles in pain. Here, we found that the protein and mRNA levels of TRPV1 and CGRP were upregulated in PIPN mice of both sexes, which indicated that TRPV1 and CGRP are associated with the development of PIPN. To explore the mechanism by which PD‐L1 affects the analgesic effect of PIPN, we found that the increase of TRPV1 and CGRP protein and mRNA levels induced by PTX were partially blocked following PD‐L1 treatment. Meanwhile, PD‐L1 and TRPV1 partially colocalize in the dorsal horn of the mouse spinal cord, which is consistent with findings from previous studies.^24^ According to our findings, TRPV1 and CGRP are partly involved in the analgesic effect of PD‐L1 on PIPN and other mechanisms may also be involved. In previous studies, PD‐L1 can affect MAPK signaling pathway and promote M2‐like polarization to reduce inflammatory response to achieve analgesia.^60^ PD‐L1 also promotes neutrophil extracellular traps (NETs) release by maintaining the transcriptional activity of STAT3, which is associated with inflammatory pain.^61^ This provides a direction for further studying other analgesic mechanisms of PD‐L1 for PIPN.

AAVs are genus‐dependent parvoviruses of the Parvoviridae family, and the use of these vectors is an advanced technology for gene delivery to treat all kinds of human diseases. Different serotypes of AAV have different tissue affinities for achieving targeted knockdown, and AAV9 has a high affinity for the central nervous system.^62^ To determine the necessity of TRPV1 for the analgesic effect of PD‐L1 on PIPN, we generated a TRPV1 knockdown mouse model by intrathecally injecting AAV9‐TRPV1‐RNAi.^63^ Afterward, we treated mice with PTX and PD‐L1, and the results showed that the analgesic effect of PD‐L1 on male TRPV1^−/−^ mice, as measured by the PWT and PWL, was partially blocked compared with that in control mice. Taken together, these findings lead to the new concept that the PD‐L1 regulating TRPV1 and CGRP expression plays a vital role in analgesia in PIPN, providing new insights into the development of novel therapies or preventors of PIPN.

Although most related experiments have been conducted on males, sex differences in pain have already attracted public attention. Previous studies have shown that not only the perception of pain but also the effect of pain treatment differ between the sexes. Many studies have shown that sex hormones are factors affecting pain sensitivity. For example, estradiol may sensitize females to temporomandibular disorder pain by upregulating TRPV1 expression in the hippocampus.^64^, ^65^ Additionally, in female mouse neurons, the combination of PD‐1 with PD‐L1 can induce p‐ERK, which theoretically enhances the ability of TRPV1 to exacerbate pain.^66^ Furthermore, sex differences exist in the human DRG, with an increase in the expression of calcitonin‐related polypeptide alpha, which encodes CGRP, in female pruritogen receptor‐enriched nociceptors.^67^ However, sex differences in PIPN and sex‐dependent analgesic effects of PD‐L1 mediated through TRPV1 and CGRP were previously unclear.

In our study, we first found that, in the PIPN model, the extent of allodynia and thermal hyperalgesia in female mice was more severe than that in male mice, and more importantly, the treatment effect induced by PD‐L1 was better than that in male mice, which demonstrated that females are more sensitive to pain.^64^, ^68^ Next, we revealed differences in the expression of TRPV1 and CGRP in the spinal cords of male and female mice, which was consistent with previous reports. Since TRPV1 and CGRP expression is positively correlated with pain progression, their differential expression in mice of both sexes may be attributed to sex differences in the clinical manifestations of PIPN and the analgesic effects of PD‐L1 on PIPN. The absence of differential TRPV1 expression between the sexes also abolishes the sex‐dependent analgesic effect of PD‐L1 on PIPN. This provides a theoretical mechanism for the treatment of sex‐specific pain.

## 5 CONCLUSIONS

In conclusion, the present findings demonstrated that PTX‐induced nociceptive behaviors were greater in female mice than in male mice, which was related to the difference in the expression of TRPV1 and CGRP in the spinal cords of PIPN mice of both sexes. Furthermore, the mechanism underlying the development of PIPN may involve PD‐L1 regulating TRPV1 and expression, highlighting the potential role of PD‐L1 and TRPV1/CGRP inhibitors in alleviating PIPN. Specifically, we first demonstrated the sex‐dependent treatment effect of PD‐L1 in PIPN mice, thereby offering novel insights for the development of sex‐specific therapies or strategies to prevent PIPN.

## AUTHOR CONTRIBUTIONS

Yan Cao, Wenqi Jiang, Wei Xing and Dongtai Chen conceived and designed the studies. Yan Cao, Wenqi Jiang, Liba Gei, Fang Yan, Yan Feng and Yuyan Pan performed majority of the experiments or provided experimental inputs. Xiangnan Chen, Yan Yan, Simin Lu and Yang Huang performed the analysis and interpretation of the data. Yan Cao, Qiang Li, Weian Zeng, and Dongtai Chen wrote the manuscript and all other authors revised it. All authors approved the final version of the paper.

## FUNDING INFORMATION

This work was supported by grants from the National Natural Science Foundation of China (grant 82172843 to WAZ), Basic and Applied Basic Research Foundation of Guangdong Province (grant 2021A1515220117 to WAZ), Medical Science and Technology Foundation of Guangdong Province (grant A2022028 to QL) and Tumor Anesthesia and Analgesia Committee of China Anti‐cancer Association (grant GWRX‐2023‐2 to YC).

## CONFLICT OF INTEREST STATEMENT

The authors declare no conflict of interest.

## Supporting information


Figure S1.

Figure S2.

## Data Availability

The raw data of the experiment were uploaded onto the Research Data Deposit (RDD) (https://www.researchdata.org.cn/default.aspx) with an RDD number of RDDB2024208693.
