# Supplementary figures and images for: Sex differences in PD‐L1‐induced analgesia in paclitaxel‐induced peripheral neuropathy mice depend on TRPV1‐based inhibition of CGRP
Source: CNS Neurosci Ther. 2024 Jul 3;30(7):e14829. doi: 10.1111/cns.14829 (PMC11222069; doi:10.1111/cns.14829)

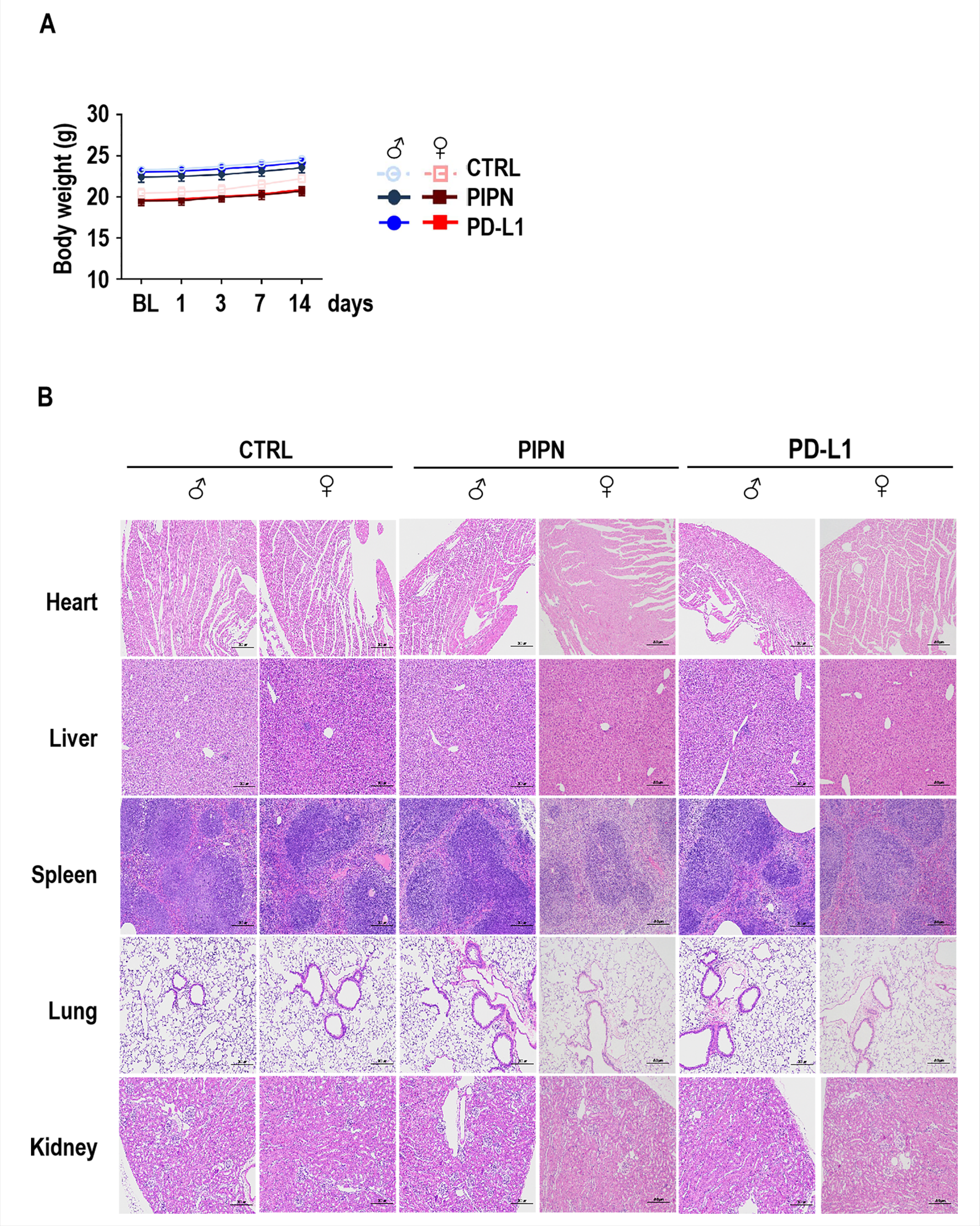

Supplement: Supplementary file 1 — Figure S1. [file CNS-30-e14829-s002.tif]

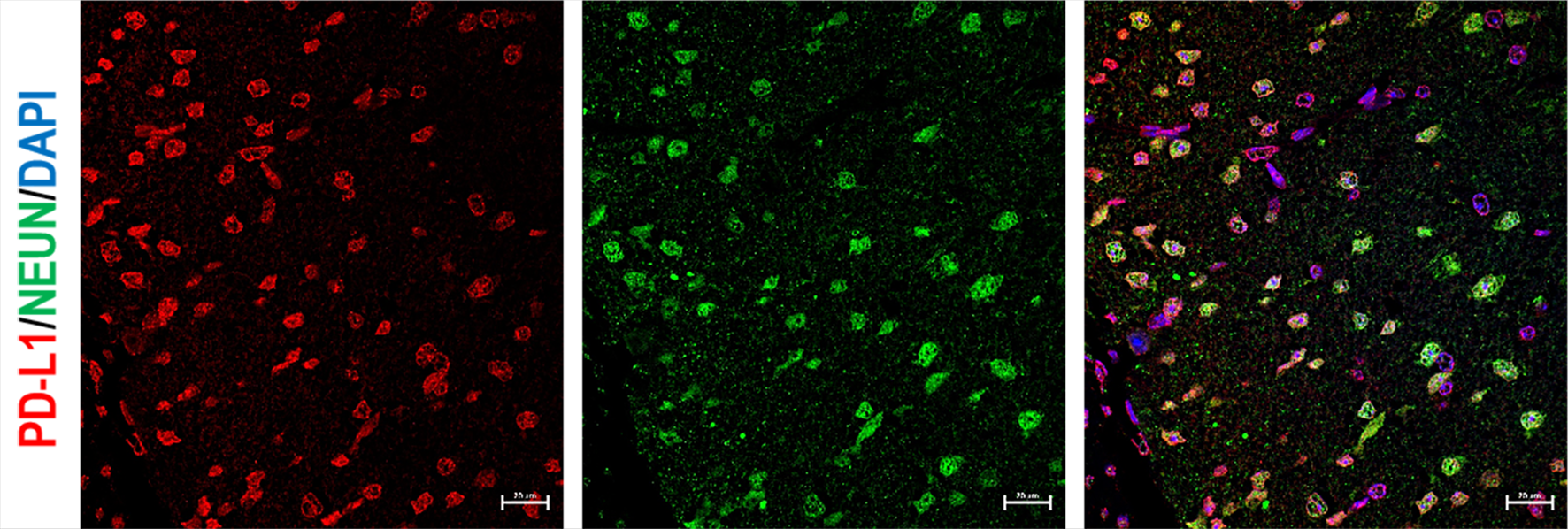

Supplement: Supplementary file 2 — Figure S2. [file CNS-30-e14829-s001.tif]
